# MCFA: Multi-Scale Cascade and Feature Adaptive Alignment Network for Cross-View Geo-Localization

**DOI:** 10.3390/s25144519

**Published:** 2025-07-21

**Authors:** Kaiji Hou, Qiang Tong, Na Yan, Xiulei Liu, Shoulu Hou

**Affiliations:** College of Computer Science, Beijing Information Science and Technology University, Beijing 102206, China; 2023020699@bistu.edu.cn (K.H.); tongq85@bistu.edu.cn (Q.T.); 2023020671@bistu.edu.cn (N.Y.); liuxiulei@bistu.edu.cn (X.L.)

**Keywords:** cross-view geo-localization, unmanned aerial vehicles (UAVs), image retrieval, remote sensing

## Abstract

Cross-view geo-localization (CVGL) presents significant challenges due to the drastic variations in perspective and scene layout between unmanned aerial vehicle (UAV) and satellite images. Existing methods have made certain advancements in extracting local features from images. However, they exhibit limitations in modeling the interactions among local features and fall short in aligning cross-view representations accurately. To address these issues, we propose a Multi-Scale Cascade and Feature Adaptive Alignment (MCFA) network, which consists of a Multi-Scale Cascade Module (MSCM) and a Feature Adaptive Alignment Module (FAAM). The MSCM captures the features of the target’s adjacent regions and enhances the model’s robustness by learning key region information through association and fusion. The FAAM, with its dynamically weighted feature alignment module, adaptively adjusts feature differences across different viewpoints, achieving feature alignment between drone and satellite images. Our method achieves state-of-the-art (SOTA) performance on two public datasets, University-1652 and SUES-200. In generalization experiments, our model outperforms existing SOTA methods, with an average improvement of 1.52% in R@1 and 2.09% in AP, demonstrating its effectiveness and strong generalization in cross-view geo-localization tasks.

## 1. Introduction

Unmanned aerial vehicles (UAVs) are widely applied in urban planning, disaster response, and remote sensing monitoring due to their flexibility and high-resolution imaging capabilities. Traditionally, drone localization relies on the Global Navigation Satellite System (GNSS) [1]. However, in urban environments, obstacles such as high-rise buildings and dense infrastructure often cause signal blockages or multipath interference, severely degrading the accuracy and reliability of GNSS-based localization. To overcome these limitations, cross-view geo-localization (CVGL) has emerged as a promising alternative. CVGL aims to estimate the location of drone images by retrieving corresponding reference images from a geotagged satellite image database despite significant viewpoint and appearance differences [2,3]. As an image retrieval problem, CVGL eliminates the dependence on GNSS and remains robust in complex or GNSS-denied environments.

Recently, deep learning’s rapid development has propelled significant progress in CVGL. Most methods learn image feature representations through neural networks, aiming to enhance discriminative capability for precise matching of positive samples and effective differentiation of negative samples. Some works [4,5] further strengthen the model’s robustness to cross-view variations through a contrastive learning strategy, which draws positive sample pairs closer together while distancing negative pairs, thus boosting the matching accuracy. However, these methods often rely heavily on global image information, neglecting the fine-grained representation of local features, which limits their effectiveness in handling complex scenes. To address this limitation, LPN [6] employs a square-ring feature segmentation strategy to capture contextual information from target-adjacent regions. FRSA [7] segments images based on semantic information and uses implicit semantic local features for image retrieval. TransFG [8] further enhances contextual correlation by performing weighted fusion of global and local features. However, despite progress in local feature learning, these methods still struggle to model relationships among local features and fail to address cross-view feature misalignment caused by differences in target orientation (e.g., buildings viewed from different angles) or imaging platforms (e.g., drones and satellites). In real-world settings, buildings are typically embedded in complex surroundings. Although the buildings themselves may lack distinctive features, their spatial relations with nearby elements provide important information for CVGL. Consequently, capturing the associations between these local features can significantly improve retrieval accuracy. Furthermore, drone imagery is captured from varying orientations and altitudes, whereas satellite imagery is typically acquired from higher altitudes with a near-vertical perspective [9]. This substantial viewpoint difference results in cross-view feature misalignment, making it difficult for cross-view images to learn consistent representations of the same target, ultimately leading to lower image retrieval accuracy. Thus, effectively modeling local relationships and addressing cross-view misalignment are key to improving cross-view geo-localization performance.

**Figure 1:**
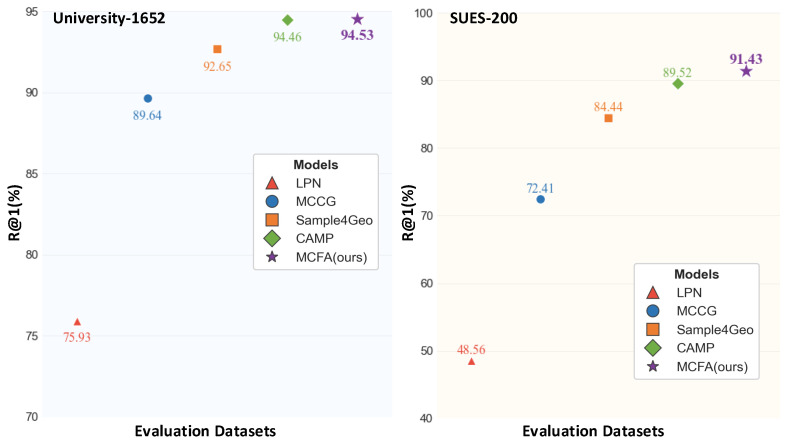
Evaluation results of our model trained on the University-1652 dataset and tested on both the University-1652 and SUES-200 datasets.

Therefore, we propose a Multi-Scale Cascade and Feature Adaptive Alignment (MCFA) network, which addresses the challenges of target representation and viewpoint inconsistency in cross-view geo-localization. Specifically, the Multi-Scale Cascade Module (MSCM) captures informative features from target-adjacent regions and integrates them with global context to enhance feature robustness. The Feature Adaptive Alignment Module (FAAM) is designed to mitigate representation misalignment between drone and satellite images. The FAAM employs a cross-dimensional dynamic weighting strategy to jointly model spatial and channel dependencies. This mechanism adaptively reweights features based on their relevance and effectively aligns cross-view representations into a unified embedding space. As shown in Figure 1, our model achieves superior performance and generalization capability compared to current SOTA methods.

The key contributions of this paper are summarized as follows:

- The MSCM enhances the model’s ability to capture and represent intricate spatial relationships between target-adjacent regions and global features, thereby improving robustness.
- The FAAM effectively adapts to feature variations of targets under different orientations and platforms, achieving cross-view feature alignment between drone and satellite images.
- Performance comparisons on benchmark datasets validate the robustness and effectiveness of our method over existing SOTA solutions. In generalization experiments, our model consistently surpasses previous SOTA approaches, achieving an average improvement of 1.74% in R@1 and 2.47% in AP, further validating its effectiveness and robustness.

## 2. Related Work

### 2.1. Cross-View Geo-Localization

Traditional works [10,11] rely on handcrafted descriptors and matching strategies, which require extensive parameter tuning and exhibiting poor generalization. Nowadays, deep learning is the mainstream approach for CVGL. Specifically, convolutional neural networks (CNNs) are employed for cross-view representation learning, demonstrating the capacity to capture semantically meaningful patterns and surpass traditional approaches [12]. Building on this, a pre-trained network was adapted through optimization of the representation discrepancy across image pairs [13], further enhancing matching accuracy. To learn discriminative feature representations, a Siamese network-based approach [14] was introduced, optimizing network parameters via contrastive loss. Additionally, the soft-margin triplet loss [15] was introduced, which encourages the reference sample to be closer to positive samples and farther from negative ones. A Siamese network incorporating NetVLAD and a weighted soft-margin ranking loss [16] was proposed to enhance training efficiency and matching accuracy. The retrieval problem was reformulated as a classification task [17] based on ResNet-50 [18]. More recently, to address the variability in drone imagery, diverse visual styles were transformed into a unified satellite image style coupled with a dynamic observation module designed to reduce surrounding noise and geographic distortions [19]. Sample4Geo [5] combined ConvNeXt [20] with contrastive learning [21,22,23] and symmetric InfoNCE loss [24], streamlining the training pipeline and enhancing generalization. MCCG [4] was based on the ConvNeXt network, capturing more discriminative information across different dimensions to obtain diverse feature representations. An Expectation-Maximization (EM)-based pseudo-label generation strategy and an information consistency module [25] were proposed to address issues such as label dependency, data association, and viewpoint differences.

### 2.2. Part-Based Feature Representation

Part-based feature representation has gained widespread application in deep learning. For example, in person re-identification (Re-ID) [26,27,28], extracting features from different body parts enhances recognition accuracy. Part-based Convolutional Baseline [29] segments pedestrian images into multiple parts, extracts features separately, and then combines them for matching. Pose-guided Part Attention [30] effectively mitigates pose variations, background clutter, and occlusions, leading to notable performance gains.

In cross-view geo-localization, LPN [6] captures contextual information from neighboring regions using a square-ring partitioning strategy, effectively emphasizing the context surrounding the target near the image center. Zhuang et al. [31] enhanced LPN by integrating two global blocks, aiming to mitigate the effects of scale changes and positional displacements. Chen et al. [32] further refined image partitioning by dividing images into finer-grained regions to address scenarios where targets are not located at the image center. Ge et al. [33] introduced a multi-branch joint representation learning network to capture both holistic image information and contextual relationships in adjacent regions. Ge et al. [34] further refined the approach by segmenting images into part-level and patch-level features as feedback to global features, introducing an adaptive region-erasing strategy to remove irrelevant information from global representations. Sun et al. [35] employed a self-supervised feature enhancement method to obtain building-aware masks, enabling the model to learn discriminative architectural regions and guide feature extraction. Wang et al. [36] proposed a decorrelation regularization approach with dynamically adjusted weights to suppress redundancy in spatial embeddings by minimizing correlations between feature channels. Zhao et al. [8] exploited the gradient characteristics of local features to strengthen cross-view feature representations and facilitate precise instance-level alignment under varying viewpoints. Liu et al. [37] proposed a two-stage learning framework, where the first stage focuses on extracting part representations and the second stage integrates these part representations into the global embedding to prevent all local parts from contributing equally to similarity measurement. Liu et al. [38] introduced graph convolutional networks to learn the semantic distribution and structural information of objects. Wu et al. [39] incorporated contrastive attribute mining and a location-aware partitioning strategy to align geographic features across different viewpoints and scales.

Despite their effectiveness, these methods often neglect the interactions among local features, which are crucial for robust cross-view representation learning. This paper proposes a part-based feature representation CVGL method called MCFA that captures complex relationships among local features and addresses feature misalignment caused by differences in orientations and platforms. MCFA consists of a Multi-Scale Cascaded Module (MSCM) and a Feature Adaptive Alignment (FAAM) module, where the MSCM integrates local and global features to enhance contextual understanding and the FAAM performs fine-grained alignment to achieve more precise cross-view feature correspondence.

## 3. Proposed Method

### 3.1. Problem Formulation

Consider a CVGL dataset containing *K* distinct geographic regions. Let the query image be denoted as $x_{j}^{k}$ and the reference image as $y_{j}^{k}$, where k∈{1,2,…,K} represents the geographic region label, and j∈{d,s} indicates the image perspective—drone view (*d*) or satellite view (*s*). The goal is to learn a feature embedding that aligns images from different viewpoints into a common space, ensuring that images from the same region are mapped close together while maintaining clear separation between those from different regions.

### 3.2. Overall Architecture

**Figure 2:**
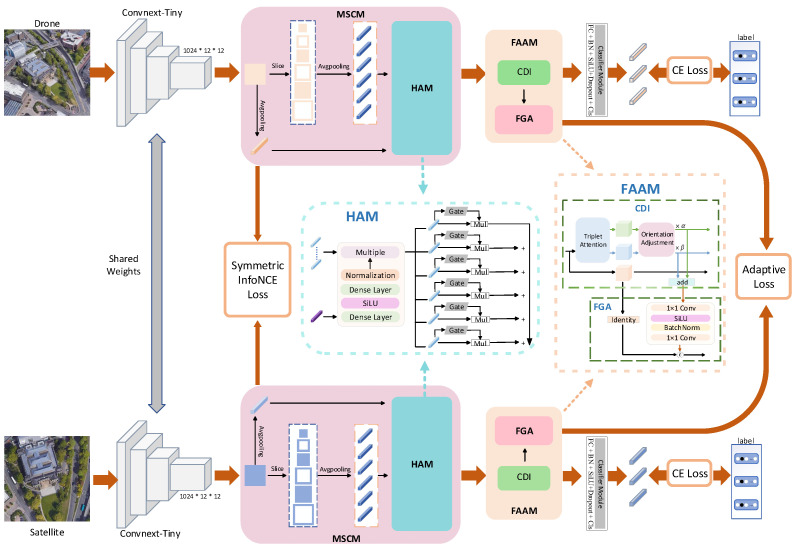
Architecture of the MCFA framework. The ConvNeXt-Tiny backbone extracts deep features from cross-view images. The MSCM enhances local–global interactions, while the FAAM dynamically adjusts spatial and channel importance for precise alignment. These components work together to improve feature representation and mitigate misalignment.

The proposed architecture comprises two parallel branches corresponding to the drone and satellite views. As illustrated in Figure 2, the framework is structured into three functional components. The first is the feature extraction module, which utilizes a pair of backbone networks with shared weights to generate feature representations from both views. The second component, the MSCM, is responsible for aggregating spatial features across multiple scales. The third component, the FAAM, refines the extracted features and aligns them to enhance cross-view correspondence.

### 3.3. Feature Extraction Module

The model employs ConvNeXt-Tiny [20] with shared weights to extract fine-grained features. ConvNeXt is a purely convolutional neural network that improves upon ResNet by incorporating architectural elements inspired by the Swin Transformer [40]. ConvNeXt-Tiny enlarges the convolution kernel to 7×7 and modifies the block stacking pattern from (3, 4, 6, 3) in ResNet to (3, 3, 9, 3). The output feature map of an input image processed by ConvNeXt-Tiny is shown as follows:

(1) $$L_{j}=F_{\mathrm{conv}}\left(x_{j}\right),\;j=d,s.$$

Here, $x_{j}$ refers to images captured from either the drone viewpoint (j=d) or the satellite viewpoint (j=s). The convolutional network $F_{\mathrm{conv}}$ extracts feature representations $L_{j}\in R^{H\times W\times C}$, where the dimensions *H*, *W*, and *C* indicate the spatial height, the spatial width, and the number of channels in the extracted feature map, respectively.

We adopt a symmetric InfoNCE loss to improve the discriminative capability of features. Specifically, compact feature vectors are generated by applying global average pooling to the feature maps $L_{j}$ extracted by ConvNeXt-Tiny, serving as inputs to the loss function. This loss pulls positive pairs closer while pushing negative pairs farther apart in the feature space, enhancing feature discriminability. It is defined as

(2) $$L_{\mathrm{InfoNCE}}\left(x_{j}^{k},y_{j}^{k}\right)=-\log\left(\frac{\exp\left(x_{j}^{k}\cdot y_{j}^{k}/\tau \right)}{\overset{R}{\underset{i=1}{\sum }}\exp\left(x_{j}^{k}\cdot y_{i}/\tau \right)}\right)$$

Here, $x_{j}^{k}$ and $y_{j}^{k}$ are the pooled feature vectors of a query image and its corresponding positive reference image. The set $\left\{y_{i}\right\}$ denotes reference features, including both positive and negative samples. When $x_{j}^{k}$ is similar to $y_{j}^{k}$, the loss value is low; otherwise, it is high. The parameter τ is a hyperparameter that dynamically adjusts during training.

### 3.4. MSCM

The proposed MSCM consists of a partitioning strategy and a Hierarchical Aggregation Module (HAM). Our partitioning strategy captures richer semantic features, while the HAM integrates global and local features, significantly enhancing feature representation.

**Figure 3:**
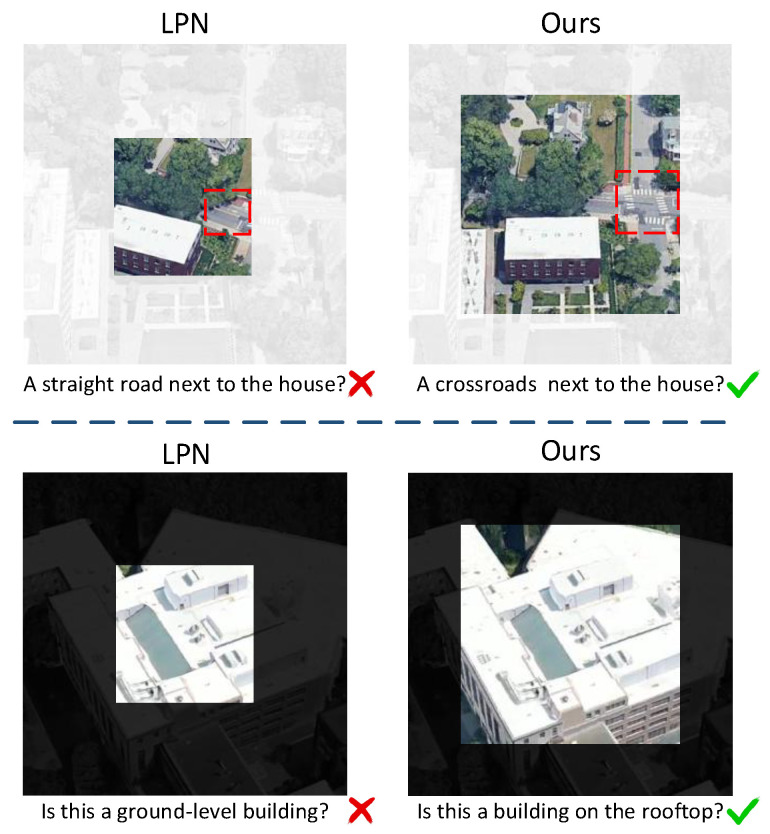
The partitioning strategy influences the understanding of the content. A smaller central partition may lead to erroneous semantic information.

**Figure 4:**
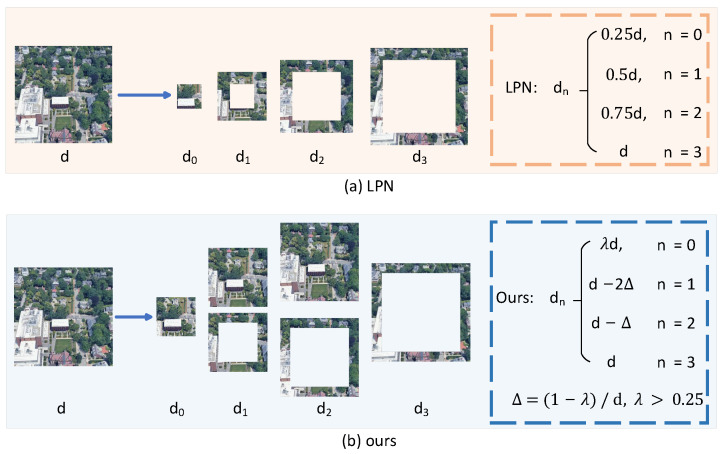
The partitioning method of LPN and ours. The first row represents LPN, while the second row represents ours.

**Partitioning strategy:** As shown in Figure 3, the original LPN [6] enhances feature discrimination but suffers from limited semantic context due to its small central region. To address this, we expand the central area to capture richer semantics and propose an improved ring-based partitioning strategy (as shown in Figure 4) that effectively captures multi-scale local information of the target and its surrounding regions, accommodating position shifts and scale variations. Specifically, the global feature $L_{j}$ (j∈{s,d}) is partitioned into *N* local features $L_{j}^{n}$ (n=1,2,…,N), which are subsequently aggregated through average pooling:

(3) $$L_{pj}^{n}=\mathrm{Pooling}\left(L_{j}^{n}\right)$$

Here, Pooling denotes average pooling, and $L_{pj}^{n}$ represents the pooled feature.

**Hierarchical Aggregation Module.** Local features provide fine-grained information, while global features represent semantic context. To fully exploit spatial information, we propose a dynamic feature fusion method that effectively integrates global features into the fusion of local features. This method adaptively adjusts the weighting between local and global features based on task requirements, enhancing the precision of local details while maintaining the coherence of global context. The fusion process significantly enhances the model’s ability to represent features and its robustness. The fusion is defined as

(4) $$L_{fj}^{n}=\mathrm{MLP}\left(\mathrm{Pooling}\left(L_{j}\right)\right)⊙L_{pj}^{n}$$

Here, the MLP block comprises a linear transformation, SiLU activation, a subsequent linear layer, and normalization. The symbol ⊙ represents element-wise multiplication. The feature $L_{fj}^{n}\in R^{H\times W\times C}$, where its dimensions are the same as $L_{j}$.

To fully explore the latent relationships between local features, we introduce a gated feature fusion method. This approach assigns adaptive weights to each local feature, enabling the model to focus on discriminative regions during the fusion process while suppressing or ignoring irrelevant areas. Specifically, each local feature is element-wise multiplied with its corresponding weight matrix, followed by linear weighting to integrate information from various local regions. This process ultimately generates a globally optimized feature representation. The formulation is as follows:

(5) $${L^{+}}_{j}=\sum_{n=1}^{N}G^{n}⊙L_{fj}^{n}$$

where ${L^{+}}_{j}\in R^{H\times W\times C}$ is the globally optimized feature representation with the same dimensions as $L_{fj}^{n}$, $G^{n}$ is the weight matrix corresponding to the *n*-th local feature, and ⊙ represents element-wise multiplication.

### 3.5. FAAM

The proposed FAAM consists of a Cross-Dimensional Interaction Mechanism (CDIM) and Fine-Grained Alignment (FGA). The CDIM adapts to feature variations across different orientations, while FGA ensures fine-grained feature alignment.

**Figure 5:**
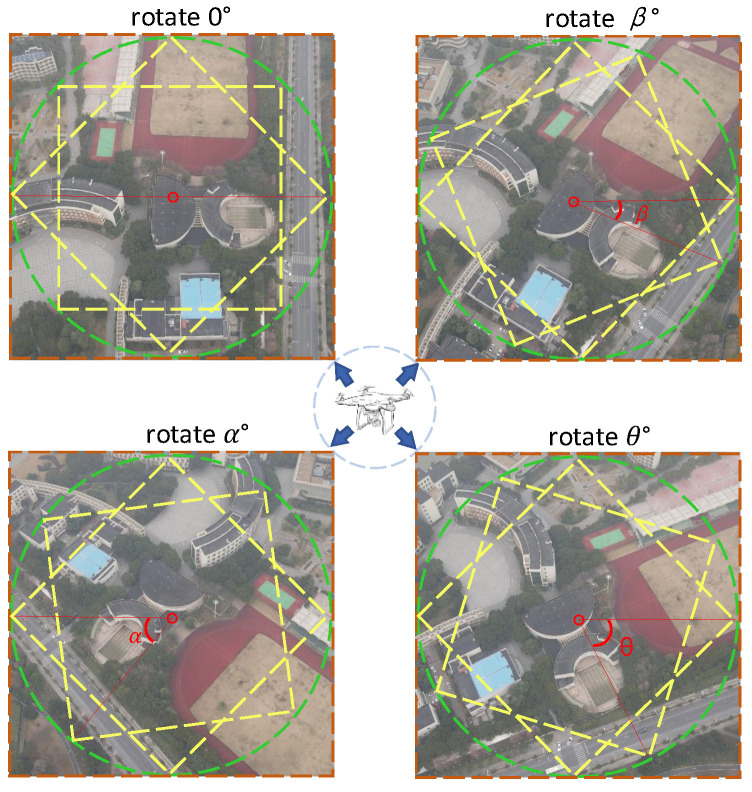
Spatial arrangement differences of the same target captured from different orientations.

**Cross-Dimensional Interaction Mechanism.** Several studies [4,41,42,43] demonstrate that incorporating attention mechanisms in both the channel and spatial dimensions can effectively enhance the model’s ability to capture critical features. Inspired by [4], we retain the first two branches of the triplet attention module. Within each branch, feature maps are transformed across spatial and channel dimensions to produce both corresponding feature maps and separate feature vectors. While both branches aim to enhance feature representation, they focus on different orientations: the first branch emphasizes the interaction between height and channels, while the second branch highlights the relationship between width and channels. However, existing methods typically assign fixed weights to these two branches, failing to account for the diverse directional arrangements that targets may exhibit under varying viewpoints or imaging conditions. Since the primary orientation of targets can be distributed at arbitrary angles (as illustrated in Figure 5), a fixed-weight strategy may limit the model’s adaptability when encountering targets with different orientations. To address this limitation, we introduce a dynamic weighting mechanism that adaptively fine-tunes the branch weights guided by the distribution of features. When the principal orientation of the target changes, this mechanism optimizes the weight allocation between the two branches, allowing the model to effectively capture critical features and adapt to targets arranged in different orientations. In this way, the dynamic weighting mechanism enhances the model’s feature representation capability, improving both the accuracy and robustness of cross-view matching. The entire procedure can be formulated as

(6) $$\left[D_{j1},D_{j2}\right]=F_{\mathrm{cross}-\mathrm{dimension}}\left({L^{+}}_{j}\right)$$

(7) $$M_{j1}=\alpha D_{j1}\cdot \frac{\exp(D_{j1})}{\exp\left(D_{j1}\right)+\exp\left(D_{j2}\right)}$$

(8) $$M_{j2}=\beta D_{j2}\cdot \frac{\exp(D_{j2})}{\exp\left(D_{j1}\right)+\exp\left(D_{j2}\right)}$$

Here, $F_{\mathrm{cross}-\mathrm{dimension}}\left(\cdot \right)$ denotes the enhanced triplet attention mechanism. The tensors $D_{j1},D_{j2},M_{j1},M_{j2}\in R^{H\times W\times C}$ represent intermediate feature maps, where $D_{j1}$ and $D_{j2}$ correspond to the outputs obtained by modeling cross-dimensional interactions between the spatial dimensions (H,W) and the channel dimension (*C*), and $M_{j1}$ and $M_{j2}$ denote the corresponding weighted features. The parameters α and β are learnable scalars used to dynamically balance the contributions of different attention branches.

**Fine-Grained Alignment.** To align cross-view image features, $M_{j1},M_{j2}$ are summed to generate the cross-dimension interaction feature $M_{j}$. This feature is then passed through an MLP to extract alignment features $f_{j}$ (j={s,d}), with dimensions $h\times w\times c_{f}$, where $c_{f}=256$. Unlike previous works [4,6], we replaced the ReLU [44] activation function with SiLU [45], whose smooth properties better handle large variations in viewpoints and scale. Next, $M_{j}$ and $f_{j}$ are concatenated in the channel dimension to form the final fused feature $F_{j}$, with dimensions $h\times w\times (c_{f}+C)$.

For improved cross-view feature alignment, we employ the cosine similarity loss function $L_{\mathrm{Cosine}}$ as an optimization objective. This loss function evaluates the alignment of feature representations to support effective cross-view matching. It is formally defined as

(9) $$L_{\mathrm{Cosine}}\left(F_{d},\;F_{s}\right)=\frac{1}{N}\sum_{i=1}^{N}\left(1-\frac{F_{d}^{i}\cdot F_{s}^{i}}{{∥F_{d}^{i}∥}_{2}{∥F_{s}^{i}∥}_{2}}\right)$$

where *N* denotes the number of feature vectors per batch, and $F_{d}^{i}$ and $F_{s}^{i}$ represent the aligned feature vectors extracted from drone and satellite images, respectively. The operator · denotes the dot product, while ${∥\cdot ∥}_{2}$ represents the L2 norm. By minimizing the cosine distance between corresponding feature vectors, this loss function effectively enhances feature alignment across different viewpoints, improving the model’s ability to establish robust and discriminative cross-view correspondences.

Building upon previous studies [4,39], we also applied global average pooling to $M_{j1}$, $M_{j2}$ and $L_{j}^{+}$ to integrate global information. The pooled features were then encoded using a classifier module to enhance their representation. The encoded features were supervised and optimized using a cross-entropy (CE) loss, thereby improving the model’s effectiveness on classification tasks. The loss function is formulated as

(10) $$\begin{array}{cc} L_{\mathrm{Classification}}\left(F,y\right) & =-\log\left(\frac{\exp(\mathrm{cls}(F,y))}{\sum_{i=1}^{N}\exp\left(\mathrm{cls}\left(F,y_{i}\right)\right)}\right) \end{array}$$

Here, the $L_{\mathrm{Classification}}$ represents the multi-class CE loss function, *F* is the feature representation generated by the classifier module, and $\hat{y}$ is the ground-truth class label for the current input.

### 3.6. Loss Function

Our loss function consists of three components and is formulated as

$$\mathrm{loss}=L_{\mathrm{InfoNCE}}+\lambda_{1}L_{\mathrm{Classification}}+\lambda_{2}L_{\mathrm{Cosine}}$$

Here, $L_{\mathrm{InfoNCE}}$ is designed to handle viewpoint differences by optimizing feature representations through contrastive learning. $L_{\mathrm{Classification}}$ supervises the optimization of encoded features via a CE loss, boosting the model’s accuracy on classification tasks. $L_{\mathrm{Cosine}}$ focuses on distinguishing hard negative samples by dynamically mining highly similar negative pairs, which facilitates the acquisition of more discriminative feature representations. $\lambda_{1}$ and $\lambda_{2}$ are adjustable coefficients that balance the influence of individual terms.

This loss function effectively balances cross-view feature alignment, hard negative sample differentiation, and classification performance optimization, enabling robust feature alignment and precise localization under diverse scenarios and conditions.

## 4. Experiments

Our experiments comprise two tasks: In the drone localization task, the drone provides the query images, and its location is determined by matching the most similar satellite image. In the drone navigation task, the satellite image is used as a query to guide the drone more accurately to the target region by retrieving the most relevant drone-view images.

### 4.1. Datasets and Evaluation Metrics

We conducted our experiments using two authoritative public datasets: University-1652 and SUES-200. Evaluation metrics are detailed in the following section.

**University-1652:** The dataset [2] comprises 1652 distinct geographic landmarks collected from 72 different universities. Each landmark contains satellite-, drone-, and street-view images, but our research focuses only on satellite and drone views. The training and test sets include 701 and 951 landmarks from 33 and 39 universities, respectively. Data acquisition leveraged Google Earth, with drone images taken at varying altitudes between 121.5 m and 256 m, providing a rich variety of multi-scale and multi-view aerial images.

**SUES-200:** The dataset [3] comprises drone and satellite images from 200 landmarks around Shanghai University of Engineering Science, with 120 used for training and 80 for testing. Each location includes 50 drone images captured at altitudes of 150 m, 200 m, 250 m, and 300 m, and 1 satellite image from AutoNavi Map and Bing Maps.

**Evaluation metrics:** To evaluate retrieval performance, we employ Recall@*K* (R@*K*) and Average Precision (AP). Recall@*K* indicates the percentage of queries with at least one correct match among the top-*K* retrievals. AP measures the overall ranking quality across recall levels. These indicators comprehensively reflect both accuracy and robustness.

### 4.2. Implementation Details

We adopted ConvNeXt-Tiny as the backbone network, initializing weights using the Kaiming initialization method [46]. Images were resized to 384 × 384 [5] and augmented via flipping, color jittering, sharpening, rotation, and occlusion.

The AdamW optimizer [47] was used during training with a batch size of 32, consisting of 16 drone and 16 satellite images. The initial learning rate was set to 0.001, with a warm-up period covering the first 10% of total training steps. The loss weighting factors $\lambda_{1}$ and $\lambda_{2}$ were assigned values of 0.1 and 0.4, respectively. All experiments were conducted on two NVIDIA RTX 3090 GPUs under PyTorch 2.5.0 on Ubuntu 22.04.

### 4.3. Comparison with SOTA Methods

#### 4.3.1. Results on University-1652

As presented in Table 1, the proposed MCFA method achieved 94.53% R@1 and 95.40% AP on the Drone→Satellite task. For the Satellite→Drone task, the model achieved 96.43% R@1 and 93.95% AP. Importantly, our approach surpasses existing SOTA techniques. Compared with CAMP, MCFA improves R@1 by 0.07% and AP by 0.02% on the Drone→Satellite task and improves R@1 by 0.28% and AP by 1.23% on the Satellite→Drone task.

#### 4.3.2. Results on SUES-200

Table 2 shows the results of our MCFA model across four different altitudes in the SUES200 dataset. The experimental results indicate that our model achieves superior performance compared to SOTA methods on the majority of evaluation metrics. Compared to CAMP, our method achieves improvements in R@1 and AP on the Drone→Satellite task at an altitude of 150 m, with R@1 and AP improving by 0.6% and 0.51%, respectively. At an altitude of 250 m, AP improves by 0.22%. In the Satellite→Drone task, our method improves R@1 and AP at 150 m by 1.25% and 1.07%, respectively. At 200 m, AP improves by 1.25%, and at 250 m, AP improves by 0.06%. Overall, MCFA demonstrates excellent performance and robust stability across various altitudes. On R@1 and AP, MCFA consistently matches or surpasses SOTA methods in multiple settings, highlighting its strong capability to handle complex spatial structures and viewpoint variations.

### 4.4. Generalization Experiments

Generalization means a model trained on one region’s data can still handle data from completely different regions. To assess the cross-domain transfer capability of our model, we trained the model on the University-1652 dataset and tested it on the SUES-200 test set. Additionally, we compared our model with current SOTA methods (MCCG, Sample4Geo, and CAMP) under the same experimental configurations. The following table summarizes the results of the generalization experiments, clearly demonstrating the excellent generalization capability of our model in the CVGL task.

Table 3 presents the evaluation results of models trained on University-1652 and tested on SUES-200. Compared to the current SOTA method CAMP, our method achieves an average improvement of 1.73% in R@1 and 2.47% in AP on the SUES-200 test set. Notably, in the 150 m height scenario, where the query and reference images exhibit significant viewpoint differences, our model outperforms CAMP by 5.75% in R@1 and 4.81% in AP for the Drone→Satellite task and by 3.75% and 7.05%, respectively, for the Satellite→Drone task. These results highlight the effectiveness of our method in handling large viewpoint variations.

However, at the 300 m altitude, our model demonstrates a slower performance improvement and slightly underperforms compared to the SOTA method. We attribute this observation to two primary factors. First, the training data employed for MCFA originates from the University-1652 dataset, which encompasses drone imagery captured at altitudes only up to 256 m. Consequently, the model was not exposed to samples from the 300 m viewpoint during training, limiting its capability to improve performance at this elevation. Second, as the acquisition altitude increases, the spatial distribution of ground-level objects such as buildings is prone to displacement toward image peripheries. This introduces heightened challenges for accurate region-level feature alignment. Despite MCFA’s incorporation of multi-scale feature fusion and adaptive alignment mechanisms designed to address such difficulties, the model’s effectiveness may still be compromised under such extreme viewpoint variations, thereby leading to a slower rate of performance enhancement.

### 4.5. Ablation Studies

A set of ablation studies was performed to further confirm the effectiveness of our proposed approach.

#### 4.5.1. Effect of the Multi-Scale Cascaded Module

The MSCM enhances the model’s capacity to capture and represent spatial features by integrating contextual information related to the target. Table 4 shows that the incorporation of this module results in a significant enhancement in model performance. In the Drone→Satellite task, R@1 increased by 1.35% and AP increased by 1.18%. In the Satellite→Drone task, R@1 improved by 1.43% and AP improved by 1.28%. These results demonstrate the superiority of the MSCM in fully mining the semantic and spatial information associations.

#### 4.5.2. Effect of the Feature Adaptive Alignment Module

The FAAM aligns and optimizes cross-view features by dynamically reweighting their significance across channels and projecting them into a unified spatial domain. Table 4 shows that after adding the Feature Adaptive alignment Module, the model achieved a 1.19% improvement in R@1 and a 0.96% improvement in AP for the Drone→Satellite task. In the Satellite→Drone task, R@1 improved by 0.85% and AP increased by 1.32%. The notable enhancement in performance demonstrates the efficiency and advantage of the FAAM in addressing the issue of cross-view feature misalignment.

#### 4.5.3. Effect of the CDIM

To validate the effectiveness of our proposed CDIM, we conduct ablation experiments by comparing it with two widely adopted attention modules: SE [48] and CBAM [42].

As shown in Table 5, the CDIM consistently outperforms both SE and CBAM across the Drone→Satellite and Satellite→Drone tasks on the University-1652 dataset. Specifically, the CDIM achieves an R@1 of 94.53% and AP of 95.40% in the Drone→Satellite task, surpassing SE by 0.95% and 0.84% and outperforming CBAM by 1.84% and 1.59%, respectively. In the Satellite→Drone task, CDIM achieves an R@1 of 96.43% and AP of 93.95%, improving upon SE by 1.14% and 1.11% and upon CBAM by 1.42% and 1.84%.

The superior performance of our CDIM stems from its focus on modeling interactions across channels and its adaptive adjustment of attention weights along both horizontal and vertical directions based on the spatial distribution of targets. This design enables the module to effectively handle variations of objects under different orientations and learn more robust invariant features.

#### 4.5.4. Effect of SiLU Activation in FGA

In the FGA, we adopt the SiLU activation function [45] in place of the commonly used ReLU [44]. To verify the impact of this design choice, we conducted ablation experiments comparing ReLU and SiLU within the FGA module. As shown in Table 6, the model with SiLU achieves superior performance on both CVGL tasks. In the Drone→Satellite direction, the model equipped with SiLU achieves a R@1 of 94.53% and AP of 95.40%, compared to 93.94% and 94.95% with ReLU. In the Satellite→Drone task, SiLU again shows improvements, achieving a R@1 of 96.43% and AP of 93.95%, outperforming ReLU’s 96.14% and 93.45%.

These results confirm that SiLU’s smooth activation curve helps stabilize feature propagation and enhances the discriminability of learned representations. By mitigating the gradient sparsity problem commonly encountered with ReLU, SiLU improves the model’s ability to adapt to continuous spatial transformations and appearance shifts across views.

#### 4.5.5. Backbone Selection and Efficiency Analysis

To comprehensively assess the trade-off between computational efficiency and retrieval performance, we evaluate two variants of our MCFA framework with different backbone architectures (ConvNeXt and ViT) and compare them against two widely adopted baseline methods, LPN [6] and Sample4Geo [5]. As summarized in Table 7, we report inference latency per image, model parameter count, and retrieval metrics on both Drone→Satellite and Satellite→Drone tasks. This comparison allows us to analyze the backbone compatibility within MCFA and benchmark its efficiency and accuracy against established baseline methods.

Our MCFA with a ConvNeXt backbone achieves the best overall retrieval performance among all compared methods, reaching 94.53% R@1 and 95.40% AP on the Drone→Satellite task and 96.43% R@1 and 93.95% AP on the Satellite→Drone task. Compared to Sample4Geo, our model not only improves retrieval accuracy but also achieves comparable runtime (3.75 ms vs. 3.64 ms) despite having a slightly larger parameter count (97.57 M vs. 87.57 M). This favorable accuracy–efficiency trade-off is largely attributable to our inference pipeline, which deploys the MSCM and FAAM only during training while relying solely on the backbone during inference.

MCFA with a ViT backbone can still achieve competitive retrieval performance compared to several mainstream methods, demonstrating the strong generalization capability of our framework across different backbone architectures. However, MCFA with a ViT backbone consumes significantly more computation time (8.80 ms) due to the inherently high computational cost of the ViT architecture. Meanwhile, both LPN and Sample4Geo have been widely deployed in practical cross-view geo-localization systems, serving as reliable baselines. Our method outperforms LPN in all key metrics with faster inference (3.75 ms vs. 4.68 ms) and surpasses Sample4Geo in both retrieval accuracy and generalization while maintaining comparable runtime, making it a strong candidate for real-world applications that require both precision and efficiency.

#### 4.5.6. Effect of Image Offset in Test Data

**Figure 6:**
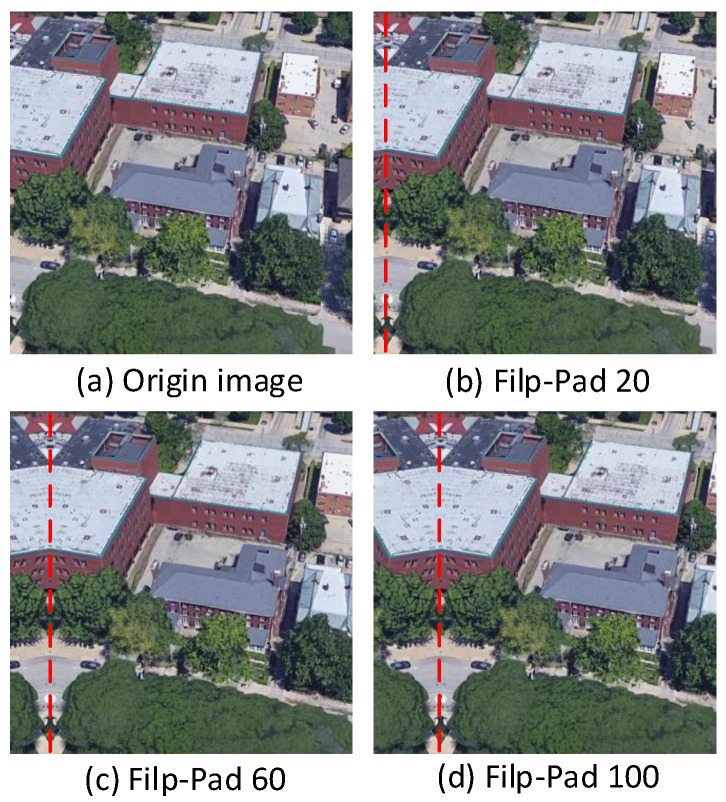
Illustration of mirror padding. The rightmost part of the image is cropped, and the leftmost region of the same width is mirrored to the left to maintain the original image size. (**a**) Original drone image; (**b**) left-side mirror padding of 20 pixels; (**c**) left-side mirror padding of 60 pixels; (**d**) left-side mirror padding of 100 pixels.

In practical applications, there is often a position discrepancy between the query image and the true matching image in the gallery. To evaluate the robustness of our model to positional deviations, we designed a mirror padding (Flip-Pad) experiment. Specifically, as illustrated in Figure 6, the mirror padding operation takes a region of width *P* from one side of the image, flips it, and attaches it back to the same side’s edge, at the same time eliminating a region of the same width from the opposite side.

**Figure 7:**
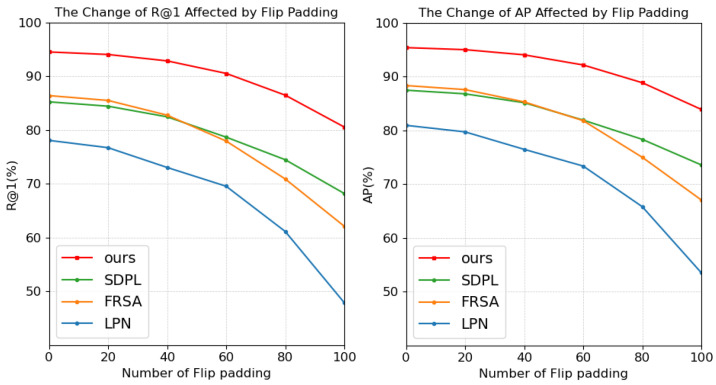
Performance comparison of our model with SDPL, FRSA, and LPN under different mirror padding settings. Our model is represented by the blue line.

**Figure 8:**
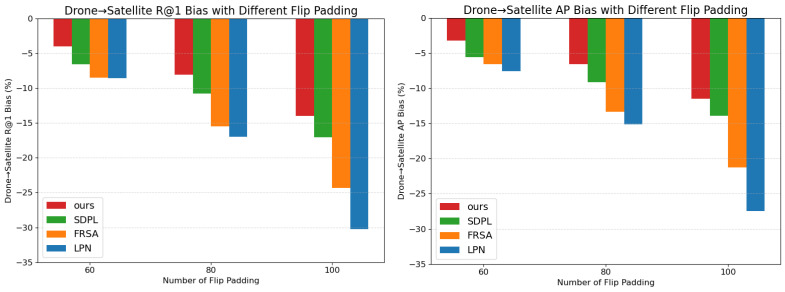
Comparison of performance degradation of our model and SDPL, FRSA, and LPN under different mirror padding settings. The blue bar in the histogram represents our model’s performance.

Table 8 provides a comparative analysis of models designed for local region extraction, including SDPL, FRSA, LPN, and our proposed model, under different offset values (P=20,40,60,80,100), where the left edge of the image was padded by mirroring. The corresponding outcomes are illustrated in Figure 7 and Figure 8. With the increase in *P*, our model exhibits a noticeably slower decline in performance relative to other models and consistently maintains superior results.

#### 4.5.7. Impact of Center Region Size on Different Cutting Methods

We investigated the effect of center region ($d_{0}$ in Figure 4) size on the model’s performance under various partitioning strategies. We partitioned the 12×12×1024 features output by ConvNeXt-Tiny, with the center region size varying from 3×3×1024 to 7×7×1024. As shown in Table 9, when the number of partitions is four, gradually increasing the width and height of the center region from 3×3 (as used in LPN) to 6×6 (half of the original image) leads to performance improvements in both two tasks. However, when the center region exceeds half of the original image size (6×6), performance declines significantly. We attribute this to the fact that a moderately sized center region allows the model to capture more complete target information, whereas an excessively large center region introduces much irrelevant information, leading to performance degradation.

To further explore this phenomenon, we increased the number of partitions to 6. As presented in Table 10, the configuration with a center region size of 4×4 (one-third of the original image) yields the best performance. Further increasing or decreasing the center region size results in performance drops, with a notable decline when the size exceeds half of the original image. This further confirms the significant impact of center region size on model performance.

#### 4.5.8. Selection of the Number of Segments

The partitioning strategy of local regions directly influences the receptive field of each part, and different region selection strategies significantly impact the model’s feature learning performance. The number of local regions *n* is a key factor in determining model performance. Table 11 presents the experimental results of the annular segmentation method using our partitioning strategy with different values of *n* on the University-1652 dataset. The results indicate that our proposed region selection method achieves optimal performance when *n* = 6. If n is too small, the model may fail to capture sufficient local features, leading to inadequate feature representation. Conversely, if n is too large, excessive noise may be introduced, reducing generalization ability. These findings demonstrate that a well-designed region partitioning strategy can effectively enhance both feature extraction capability and model robustness.

#### 4.5.9. Comparison of Different Loss Functions on Fine-Grained Alignment

To investigate the impact of various loss functions on Fine-Grained Alignment (FGA), we conducted experiments on the University-1652 dataset. As shown in Table 12, the superiority of cosine similarity loss lies in its direct optimization of the angular relationship between feature vectors rather than their absolute magnitudes, making it inherently scale-invariant and more effective in aligning features across different viewpoints. In contrast, MSE and triplet loss rely on Euclidean distance, which is sensitive to variations in feature magnitudes. In cross-view scenarios, perspective distortion and scale differences often lead to unstable feature magnitudes, ultimately degrading retrieval performance. InfoNCE loss tends to blur the semantic boundary between positive and negative samples for the complex features produced by FGA, increasing the risk of misclassifying hard positives as negatives and thereby weakening the model’s discriminative power and overall matching performance.

### 4.6. Model Visualization

**Figure 9:**
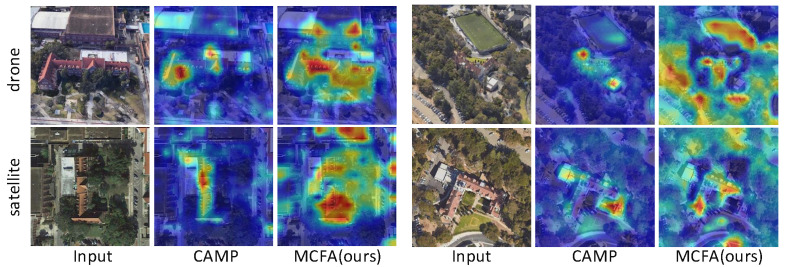
Comparison of heatmaps between our proposed method and the existing CAMP model for both drone and satellite view images on University-1652.

Figure 9 displays the heatmaps obtained from our model and the comparison model CAMP from both drone and satellite perspectives. Compared to CAMP, our model accurately captures the key regions of the target as well as the surrounding meaningful contextual areas. This further demonstrates that during feature extraction, our approach enhances the model’s sensitivity to target-specific information, significantly improving the precision and robustness of cross-view geo-localization.

**Figure 10:**
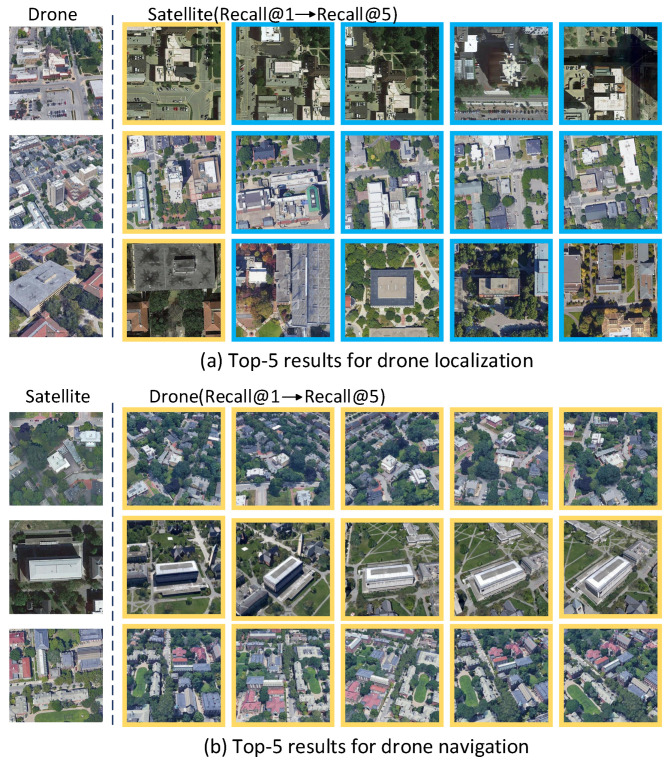
Top-5 retrieval results for (**a**) drone localization and (**b**) drone navigation. The yellow box indicates a correct match, while the blue box indicates an incorrect match.

To validate the effectiveness of our model in drone localization and navigation tasks, we randomly selected three images from the query_drone and query_satellite subsets of the University-1652 test set for testing. For each query image, we retrieved the top-five matching images. Figure 10 presents the retrieval results, showing that our model exhibits excellent retrieval accuracy in drone localization and navigation tasks.

## 5. Limitation

### 5.1. Feature Separability and Failure Case Analysis

**Figure 11:**
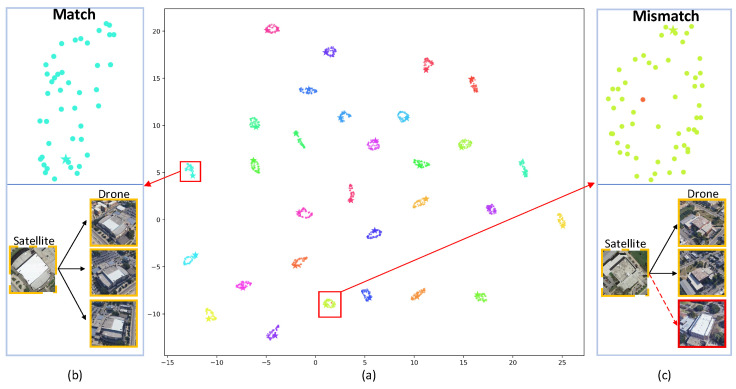
t-SNE visualization of feature distributions. (**a**) shows the global view of feature embeddings. (**b**) highlights successful matches between drone and satellite features. (**c**) presents mismatches caused by high visual similarity between different scenes, where red dashed lines indicate incorrect matches.

Figure 11a presents the t-SNE [50] visualization results of our model. As shown in Figure 11b, the features are well clustered, indicating successful matching. In contrast, Figure 11c illustrates a failure case, where the drone image is mismatched due to the high visual similarity between two different scenes. The model struggles to distinguish subtle differences, resulting in incorrect associations.

### 5.2. Robustness Analysis Under Illumination Variations

**Figure 12:**
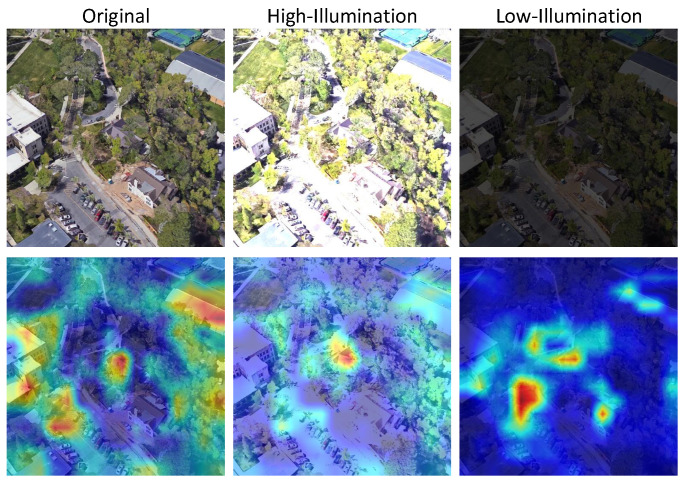
Heatmaps under varying illumination conditions on University-1652.

To further analyze the robustness of our model under varying illumination conditions, we visualize the attention heatmaps generated from three types of input: original images, processed high-illumination images, and processed low-illumination images. As shown in Figure 12, the model performs well under normal lighting, accurately focusing on discriminative regions such as roads and rooftops. However, under strong illumination, overexposure leads to the disappearance of key structures—roads become barely visible, and rooftops are difficult to distinguish from the surrounding environment. In low-light scenarios, buildings tend to merge with the background, resulting in suppressed attention responses and reduced feature separability. These observations suggest that extreme lighting conditions significantly degrade the model’s ability to extract informative spatial features.

### 5.3. Analysis at 300 m Altitude in Generalization Setting

**Figure 13:**
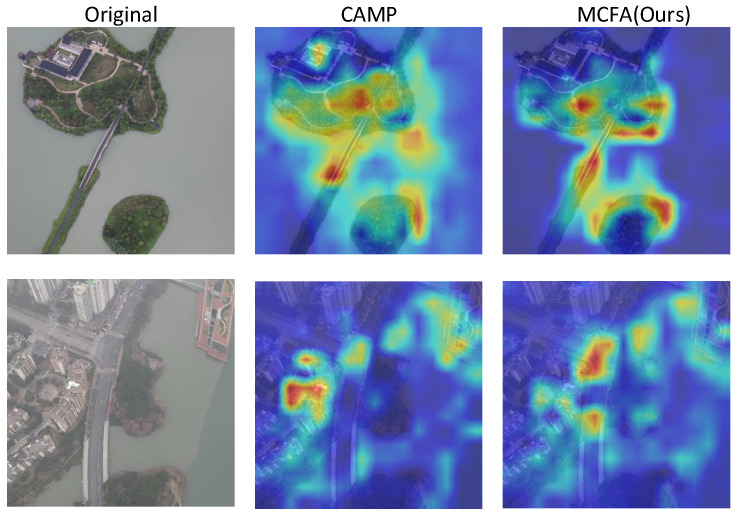
Heatmaps at 300 m altitude on SUES-200.

As shown in Table 3, our MCFA model achieves superior generalization performance across most altitudes in both Drone→Satellite and Satellite→Drone directions when trained on University-1652 and evaluated on SUES-200. However, at the 300-meter altitude, CAMP slightly outperforms our model. To investigate this behavior, we visualize representative samples at 300 m in Figure 13. It can be observed that buildings exhibit noticeable spatial shifts, often appearing near the image periphery rather than near the center. This spatial displacement poses a significant challenge for region-level alignment. Although MCFA is equipped with multi-scale feature fusion and adaptive alignment modules, such extreme viewpoint variations can still restrict its ability to accurately localize and match target regions. As a result, MCFA shows limited performance improvement at 300 m compared to its strong gains at lower altitudes. Furthermore, as shown in Figure 13, our model tends to focus more on road-related structures under high-altitude conditions, whereas CAMP, benefiting from its dynamic positional encoding, adapts more effectively to building displacement. This results in CAMP achieving a slight advantage under this specific extreme setting. Nonetheless, our model remains competitive across most conditions and performs robustly in general.

## 6. Conclusions

To deeply explore the contextual information of the target and effectively address the issue of cross-view image feature misalignment, this paper proposes the MCFA network. This model integrates an MSCM, which captures rich contextual information by learning deep connections between neighboring regions of the target, and an FAAM, which adaptively adjusts feature importance to enhance fine-grained feature representation and achieve accurate cross-view alignment. In terms of experimental evaluation, the proposed model demonstrates leading performance on standard datasets such as University-1652 and SUES-200, significantly surpassing existing baselines in R@1 and AP metrics and outperforming SOTA methods in generalization tests, fully validating the effectiveness and superiority of our model in cross-view geo-localization tasks. We plan to further optimize the model, particularly in the fine-grained feature mining of target neighboring regions, to enhance the model’s robustness in complex environments. We hope to advance the development of cross-view geo-localization through these efforts, especially in challenging scenarios such as illumination changes and large-scale variations.

## Figures and Tables

**Table 1:** Comparison with SOTA results on University-1652.

Method	Publication	Drone→Satellite		Satellite→Drone	
		R@1	AP	R@1	AP
Instance Loss [2]	ACM MM’2020	59.69	64.80	73.18	59.40
LPN [6]	TCSVT’22	75.93	79.14	86.45	74.79
FSRA [7]	TCSVT’22	82.25	84.82	87.87	81.53
TransFG [8]	TGRS’24	84.01	86.31	90.16	84.61
SeGCN [38]	JSTARS’24	89.18	90.89	94.29	89.65
MCCG [4]	TSCVT’23	89.64	91.32	94.30	89.39
SDPL [32]	TSCVT’24	90.16	91.64	93.58	89.45
Sample4Geo [5]	ICCV’23	92.65	93.81	95.14	91.39
CAMP [39]	TGRS’24	94.46	95.38	96.15	92.72
MCFA (ours)		**94.53**	**95.40**	**96.43**	**93.95**

The best results are in bold.

**Table 2:** Comparison with SOTA results on SUES-200.

Drone→Satellite									
**Method**	**Publication**	**150 m**		**200 m**		**250 m**		**300 m**	
		**R@1**	**AP**	**R@1**	**AP**	**R@1**	**AP**	**R@1**	**AP**
SUES-200 Baseline [3]	TCSVT’23	55.65	61.92	66.78	71.55	72.00	76.43	74.05	78.26
LPN [6]	TCSVT’22	61.58	67.23	70.85	75.96	80.38	83.80	81.47	84.53
MCCG [4]	TCSVT’23	82.22	85.47	89.38	91.41	93.82	95.04	95.07	96.20
SDPL [32]	TCSVT’24	82.95	85.82	92.73	94.07	96.05	96.69	97.83	98.05
SeGCN [38]	JSTARS’24	90.80	92.32	91.93	93.41	92.53	93.90	93.33	94.61
Sample4Geo [5]	ICCV’23	92.60	94.00	97.38	97.81	**98.28**	98.64	99.18	99.36
CAMP [39]	TGRS’24	95.40	96.38	**97.63**	**98.16**	98.05	98.45	99.23	**99.46**
MCFA (ours)		**96.00**	**96.89**	97.38	97.86	98.25	**98.67**	**99.28**	99.42
**Satellite→Drone**									
**Method**	**Publication**	**150 m**		**200 m**		**250 m**		**300 m**	
		**R@1**	**AP**	**R@1**	**AP**	**R@1**	**AP**	**R@1**	**AP**
SUES-200 Baseline [3]	TCSVT’23	75.00	55.46	85.00	66.05	86.25	69.94	88.75	74.46
LPN [6]	TCSVT’22	83.75	66.78	88.75	75.01	92.50	81.34	92.50	85.72
MCCG [4]	TCSVT’23	93.75	89.72	93.75	92.21	96.25	96.14	98.75	96.64
SDPL [32]	TCSVT’24	93.75	83.75	96.25	92.42	97.50	95.65	96.25	96.17
SeGCN [38]	JSTARS’24	93.75	92.45	95.00	93.65	96.25	94.39	97.50	94.55
Sample4Geo [5]	ICCV’23	**97.50**	93.63	**98.75**	96.70	**98.75**	**98.28**	98.75	98.05
CAMP [39]	TGRS’24	96.25	93.69	97.50	**96.76**	**98.75**	98.10	**100.00**	**98.85**
MCFA (ours)		**97.50**	**94.76**	**98.75**	96.60	**98.75**	98.16	98.75	98.68

The best results are in bold.

**Table 3:** Generalization experiment when trained on the UNIVERSITY-1652 dataset and evaluated on SUES-200.

Drone→Satellite								
**Method**	**150 m**		**200 m**		**250 m**		**300 m**	
	**R@1**	**AP**	**R@1**	**AP**	**R@1**	**AP**	**R@1**	**AP**
LPN [6]	32.85	40.10	43.80	50.67	49.75	56.55	54.10	60.73
MCCG [4]	57.62	62.80	66.83	71.60	74.25	78.35	82.55	85.27
Sample4Geo [5]	74.70	78.47	81.28	84.40	86.88	89.28	89.28	91.24
CAMP [39]	76.53	80.47	87.18	89.60	93.75	95.04	**96.40**	**97.18**
MCFA (ours)	**82.38**	**85.28**	**90.70**	**92.21**	**94.38**	**95.24**	95.30	96.00
**Satellite→Drone**								
**Method**	**150 m**		**200 m**		**250 m**		**300 m**	
	**R@1**	**AP**	**R@1**	**AP**	**R@1**	**AP**	**R@1**	**AP**
LPN [6]	32.50	26.60	40.00	35.10	46.50	41.88	53.50	48.47
MCCG [4]	61.25	53.51	82.50	67.06	81.25	74.99	87.50	80.20
Sample4Geo [5]	82.50	76.20	85.00	82.93	92.50	87.77	92.50	88.38
CAMP [39]	88.75	78.17	95.00	88.31	95.00	91.85	96.25	93.43
MCFA (ours)	**92.50**	**85.22**	**95.00**	**91.46**	**95.00**	**93.46**	**97.50**	**94.93**

The best results are in bold.

**Table 4:** The results of ablation studies with MSCM and FAAM on University-1652.

Method	Drone→Satellite		Satellite→Drone	
	**R@1**	**AP**	**R@1**	**AP**
w/o both	91.96 +0.00	93.25 +0.00	94.15 +0.00	90.86 +0.00
w/MSCM	93.31 +1.35	94.43 +1.18	95.58 +1.43	92.14 +1.28
w/FAAM	93.15 +1.19	94.21 +0.96	95.00 +0.85	92.18 +1.32
MCFA (ours)	**94.53** +2.57	**95.40** +2.15	**96.43** +2.28	**93.95** +3.09

The best results are in bold.

**Table 5:** Comparison of different attention modules on University-1652.

Method	Drone→Satellite		Satellite→Drone	
	**R@1**	**AP**	**R@1**	**AP**
SE [48]	93.58	94.56	95.29	92.84
CBAM [42]	92.69	93.81	95.01	92.11
CDIM (ours)	**94.53**	**95.40**	**96.43**	**93.95**

The best results are in bold.

**Table 6:** Performance comparison of activation functions in the FGA module on University-1652.

Activation Function	Drone→Satellite		Satellite→Drone	
	**R@1**	**AP**	**R@1**	**AP**
ReLU [44]	93.94	94.95	96.14	93.45
SiLU [45]	**94.53**	**95.40**	**96.43**	**93.95**

The best results are in bold.

**Table 7:** Efficiency and performance comparison of different methods on University-1652.

Method	Runtime	Params	Drone→Satellite		Satellite→Drone	
	**(ms)**	**(M)**	**R@1**	**AP**	**R@1**	**AP**
LPN [6]	4.68	58.29	75.93	79.14	86.45	74.79
Sample4Geo [5]	3.64	87.57	92.65	93.81	95.14	91.39
MCFA (ViT)	8.80	85.51	88.73	90.66	95.01	92.11
MCFA (ConvNeXt)	3.75	97.57	**94.53**	**95.40**	**96.43**	**93.95**

The best results are in bold.

**Table 8:** The effect of image offset.

Offset	MCFA (Ours)		SDPL [32]		FRSA [7]		LPN [6]	
	**R@1**	**AP**	**R@1**	**AP**	**R@1**	**AP**	**R@1**	**AP**
0	94.53 −0.00	95.40 −0.00	85.25 −0.00	87.48 −0.00	86.41 −0.00	88.34 −0.00	78.08 −0.00	80.94 −0.00
+20	94.06 −0.47	95.02 −0.38	84.44 −0.81	86.80 −0.68	85.51 −0.90	87.59 −0.75	76.72 −1.36	79.72 −1.22
+40	92.87 −1.66	94.06 −1.34	82.46 −2.79	85.15 −2.33	82.77 −3.64	85.30 −3.04	73.04 −5.04	76.47 −4.47
+60	90.54 −3.99	92.17 −3.23	78.68 −6.57	81.91 −5.57	77.95 −8.46	81.18 −7.16	69.54 −8.54	73.36 −7.58
+80	86.49 −8.04	88.87 −6.53	74.48 −10.77	78.33 −9.15	70.90 −15.51	74.99 −13.35	61.13 −16.95	65.80 −15.14
+100	80.56 −13.97	83.91 −11.49	68.19 −17.06	73.57 −13.91	62.10 −24.31	67.05 −21.29	47.87 −30.21	53.50 −27.44

**Table 9:** Impact of center region size on different cutting methods when number = 4.

Size	Satellite→Drone		Drone→Satellite	
	**R@1**	**AP**	**R@1**	**AP**
3×3	92.72 +0.00	93.88 +0.00	95.29 +0.00	92.40 +0.00
4×4	93.84 +1.12	94.80 +0.92	96.15 +0.86	93.26 +0.86
5×5	93.66 +0.94	94.64 +0.76	**96.29** +1.00	**93.34** +0.94
6×6	**93.89** +1.17	**94.82** +0.94	95.58 +0.29	92.94 +0.54
7×7	92.34 −0.38	93.51 −0.37	95.15 −0.14	91.99 −0.41

The best results are in bold.

**Table 10:** Impact of center region size on different cutting methods when number = 6.

Size	Satellite→Drone		Drone→Satellite	
	**R@1**	**AP**	**R@1**	**AP**
3×3	93.84 +0.00	94.82 +0.00	95.58 +0.00	93.34 +0.00
4×4	**94.53** +0.69	**95.40** +0.58	**96.43** +0.85	**93.95** +0.61
5×5	94.03 +0.19	94.97 +0.15	96.15 +0.57	93.59 +0.25
6×6	93.88 +0.04	94.87 +0.05	96.15 +0.57	93.24 −0.10
7×7	92.42 −1.42	93.60 −1.22	95.44 −0.14	91.99 −1.35

The best results are in bold.

**Table 11:** The results of ablation studies with different numbers of cut regions.

Number	Drone→Satellite		Satellite→Drone	
	**R@1**	**AP**	**R@1**	**AP**
Ours (4)	93.26	94.34	95.72	92.73
Ours (6)	**94.53**	**95.40**	**96.43**	**93.95**
Ours (8)	92.44	93.58	95.72	92.47
Ours (10)	92.46	93.63	95.29	92.30

The best results are in bold.

**Table 12:** Performance of FGA with different loss functions.

Loss Function	Satellite→Drone		Drone→Satellite	
	**R@1**	**AP**	**R@1**	**AP**
InfoNCE	91.65	93.02	94.72	90.78
MSE loss	93.09	94.15	95.58	92.18
Triplet loss	93.21	94.25	94.86	92.09
Hard Triplet Loss [49]	94.13	95.04	95.72	93.57
Cosine Similarity Loss	**94.53**	**95.40**	**96.43**	**93.95**

The best results are in bold.

## Data Availability

The public sources of the data mentioned in this study are described in the paper.
